# Novel *Mammalian orthorubulavirus 5* Discovered as Accidental Cell Culture Contaminant

**DOI:** 10.3390/v11090777

**Published:** 2019-08-23

**Authors:** Brandi J. Feehan, Aleksey A. Penin, Alexey N. Mukhin, Deepak Kumar, Anna S. Moskvina, Kizkhalum M. Khametova, Anton G. Yuzhakov, Maria I. Musienko, Alexey D. Zaberezhny, Taras I. Aliper, Douglas Marthaler, Konstantin P. Alekseev

**Affiliations:** 1 Veterinary Diagnostic Laboratory, College of Veterinary Medicine, Kansas State University, Manhattan, KS 66053, USA; 2 Institute for Information Transmission Problems of the Russian Academy of Sciences, Moscow 127051, Russia; 3 N. F. Gamaleya Federal Research Center for Epidemiology & Microbiology, Moscow 123098, Russia; 4 Diagnostic and Prevention Research Institute for Human and Animal Diseases, Moscow 123098, Russia; 5 Federal State Budget Scientific Institution “Fedral Scientific Centre VIEV”, Moscow 109428, Russia

**Keywords:** *Mammalian orthorubulavirus 5*, *Mammalian rubulavirus 5*, *Parainfluenza virus 5*

## Abstract

A distinct Russian *Mammalian orthorubulavirus 5* (PIV5) was detected in cell culture exhibiting cytopathic effect and hypothesized to be contaminated by a scientist with respiratory symptoms. The identification of the divergent strain indicated a lack of knowledge on the diversity of PIV5 strains and calls for surveillance of global PIV5 strains.

## 1. Introduction

*Mammalian orthorubulavirus 5* (PIV5), formerly named parainfluenza virus 5, resides within the Paramyxoviridae family [1,2,3]. The Paramyxoviridae family contains four subfamilies, Rubulavirinae, Avulavirinae, Metaparamyxovirinae, and Orthoparamyxovirinae. PIV5 is classified with seven other viruses in the *Orthorubulavirus* genus, one of two genera within Rubulavirinae [1,2]. PIV5 is globally distributed and has been associated with respiratory disease in canine, cattle, swine, and lesser panda [4,5,6,7]. While the pathogenesis of PIV5 in humans is unknown, both PIV5 and contagious *Mumps orthorubulavirus* belong to the *Orthorubulavirus* genus [3]. Symptoms of *Mumps orthorubulavirus* in humans include fever, swollen salivary glands and possibly miscarriages or hearing loss [2,8]. We discovered a divergent Russian PIV5 strain (Moskva) in Vero cells exhibiting cytopathic effect (CPE). The cells were suspected to be accidentally contaminated by an ill laboratory technician, since the samples used for inoculation and the cells were negative for Moskva by PCR. In addition, Moskva contained a small hydrophobic (SH) protein coding sequence (CDS) present in human PIV5 strains. 

## 2. The Study

**Figure 1:**
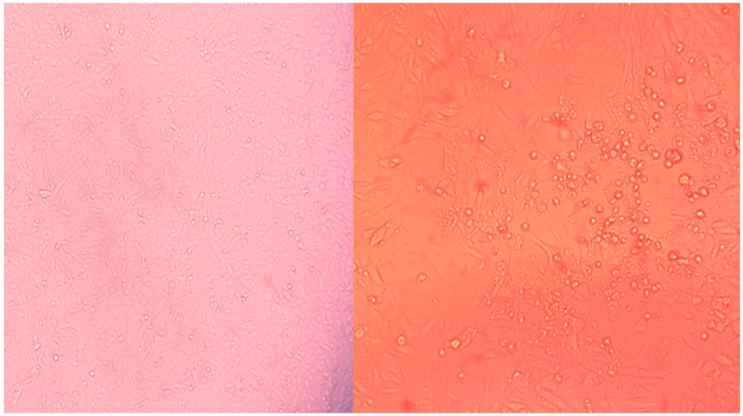
Cell cultures illustrating cytopathic effect (CPE) in the sequenced culture with Moskva (**right**) relative to the control (**left**).

In November 2015, an outbreak of enteric disease occurred in three-day-old piglets on a farm residing in the Republic of Buryatia, Russia (Supplemental Materials and Methods) [9]. Fecal sample supernatants were passaged on Vero cells in the hope of isolating RVB [9]. Concurrently, the scientist passaging the cell cultures exhibited mild respiratory symptoms including tussis and rhinorrhea. Cytopathic effect (syncytia formation) was observed on the fourth passage of the cells (Figure 1). The nucleic acid from the 15th passage was extracted, and NGS was performed to identify the CPE causative pathogen using an Illumina HiSeq2000 (Illumina, San Diego, CA, USA) [9]. NGS generated 9,280,254 paired reads while 8,949,411 reads resulted after trimming [9]. Kraken identified ~85% (7,602,876) paired reads as Eukaryotic and ~9% (818,499) as viral. Of viral reads, ~65% (534,325) were classified as Paramyxoviridae and ~33% (271,890) were classified as Retroviridae (retrovirus) [10]. *De novo* assembly of the viral reads generated a PIV5 genome (Moskva, MK593539), which upon BLASTn yielded an 87% nucleotide identity to human PIV5 strain DEN, isolated from the United Kingdom in 1980 (JQ743322). The highest BLASTn hit to a non-PIV5 strain was to a *Human orthorubulavirus 2* strain (68%) from Vietnam (MH006623). Primers (forward, CCGGATCACGTGTCCTCAAA; and reverse, ACCAGGAACCGCACTAATGG) were designed to Moskva, and a PCR was developed utilizing RevertAid H Minus First Strand cDNA Synthesis Kit using recommended manufacturer protocols (Thermo Fisher Scientific, Waltham, MA, USA). The original Vero cell stock and fecal samples were negative for Moskva by PCR. Testing of the cell culture from passage four demonstrated PIV5 titers of ~10^3.5^ TCID_50_/mL [11], supporting the hypothesis of human contamination of the cell culture.

The Moskva sequence was 15,218 nucleotides in length, within an incompletely sequenced 5’ untranslated region due to low coverage (<10 reads). Moskva contained a genome organization of nucleocapsid (N), V, phosphoprotein (P), matrix (M), fusion (F), SH, hemagglutinin-neuraminidase (HN), and large (L) genes [3]. The M protein is the most abundant capsid protein, and the L protein is crucial for RNA synthesis in paramyxoviruses [3]. Both the M and L CDS regions exhibited novel PIV5 truncations of 9 and 11 residues, respectively, which exemplified the diversity of Moskva. In addition, Moskva contained the putative SH gene. The presence of the SH gene varies amongst PIV5 strains, and six of seven human strains contain the SH gene. The SH CDS was present in Moskva, supporting the hypothesis of human contamination of the Vero cell culture [4]. The SH protein has been suggested to limit apoptosis via tumor necrosis factor α (TNFα) and causes a decrease in CPE *in vitro* [3,12]. The identified CPE and proposed function of the SH protein contradicts our observed CPE in Moskva.

**Figure 2:**
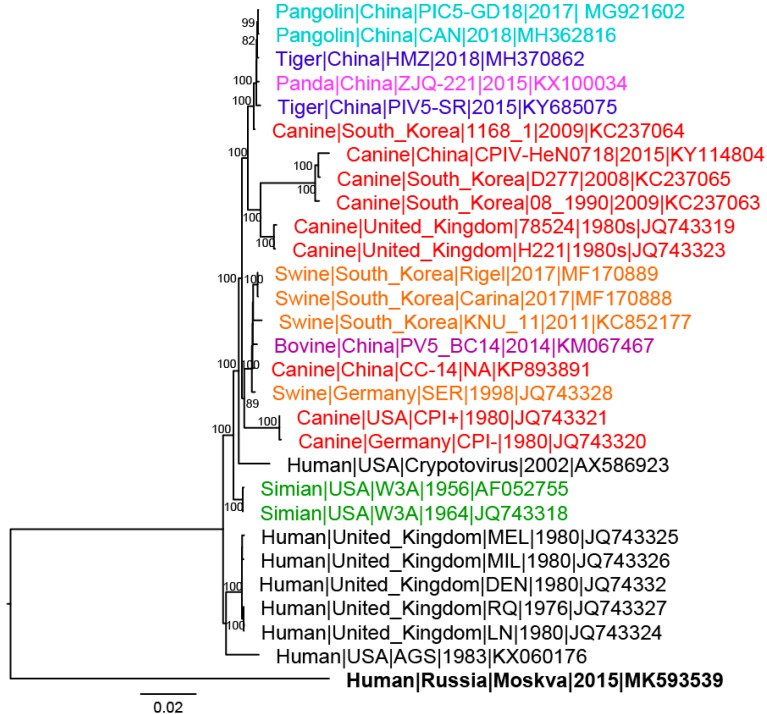
Whole genome phylogenetic tree of *Mammalian orthorubulavirus 5* (PIV5) strains. Bootstrap values greater than 70 are shown at major nodes. Strains are colored according to host with black representing human strains; red, canine; orange, swine; green, simian; teal, pangolin; blue, tiger; pink, panda; and purple, bovine. Moskva is in bold.

Pairwise nucleotide and amino acid MAFFA alignments were generated in Geneious version 11.5.1 to calculate identities (Geneious, Newark, NJ, USA). Nucleotide percent identities of the complete genome increased the diversity of the PIV5 strains in GenBank from 95.5–100% to 85.4–100% (Table 1). Using the 80% whole genome nucleotide identity classification set by the International Committee on Taxonomy of Viruses, Moskva belongs to the *Rubulavirus* genus [1,2]. Moskva demonstrated an 84.9–88.8% nucleotide identity to the other PIV5 strains in GenBank regardless of the gene or host species (Table 1), highlighting the diversity of Moskva. Moskva had a 96.9% amino acid identity of RNA dependent RNA polymerase (Large protein) to the other PIV5 strains, illustrating Moskva as a member of the PIV5 species. The divergence of Moskva was reflected as a distinct lineage in a whole genome phylogenetic tree (Figure 2). The 500 bootstrapped maximum-likelihood tree was created in Geneious and edited in FigTree (Institute of Evolutionary Biology, University of Edinburgh, Edinburgh, Scotland). Host and geographic clades were absent in the tree, which was reflected in all individual gene phylogenetic trees (Figure S1). The divergence in genomic identities and phylogenetic trees suggests undiscovered divergent PIV5 strains are circulating in geographically distinct regions.

**Figure 3:**
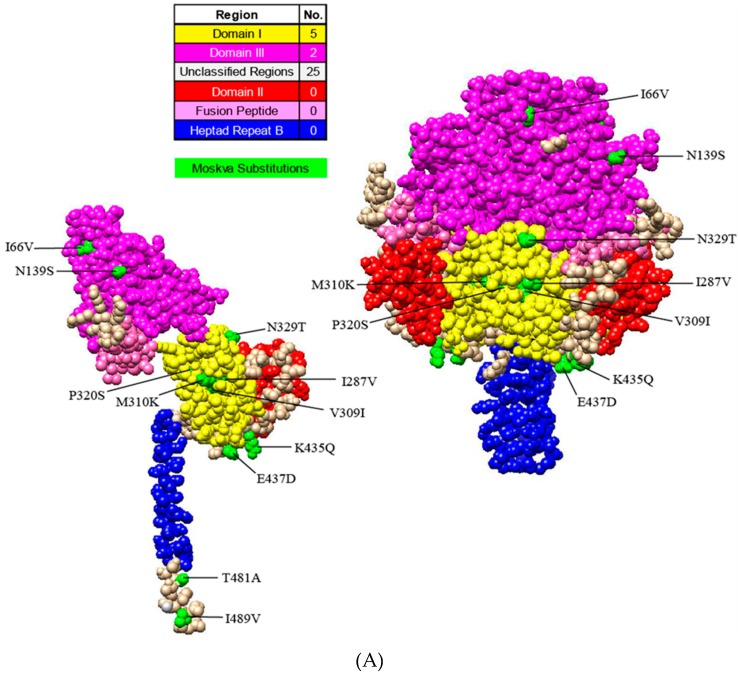

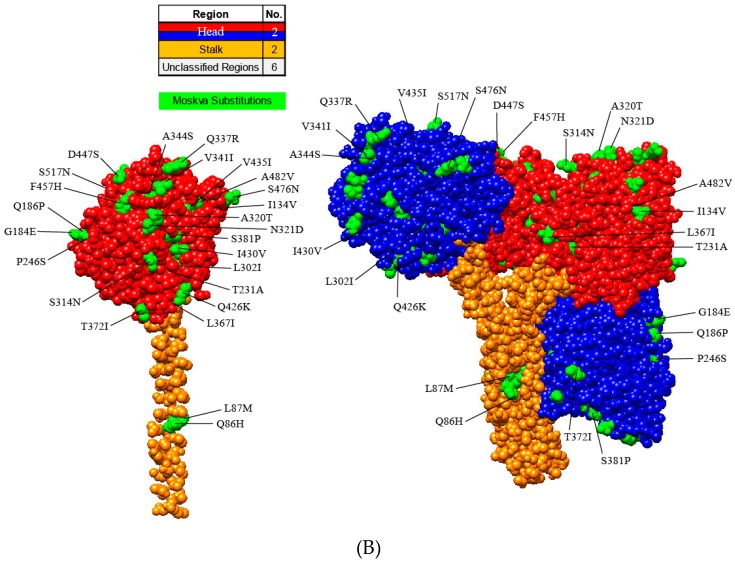
Protein models illustrating the residue substitutions in Moskva. Monomer and trimer models in uncleaved Fusion (**A**) and Hemagglutinin-neuraminidase (HN) (**B**) proteins. Regions and substitutions are colored according to the legend.

The PIV5 genome encodes two glycoproteins, F and HN, which are essential for viral attachment, fusion, and entry. With Moskva exhibiting human origin, the F and HN amino acid sequences of Moskva were compared to the F and HN of human strains. The protein models illustrated the location and magnitude of substitutions using SWISS-MODEL (Swiss Institute of Bioinformatics, Lausanne, Switzerland; F, 4jf7.1; HN, 4wsg.1), Phyre2 (Imperial College London, London, UK; F, c2b9bA; HN, c4jf7B), and Chimera (Resource for Biocomputing, Visualization and Informatics, University of California, San Francisco, CA, USA). Consistent with Moskva’s evolutionary divergence, the F and HN proteins of Moskva had more residue substitutions (32 and 31, respectively) relative to consensus sequences of human PIV5 strains (Figure 3A). Of the 32 Moskva F protein substitutions, the majority of the substitutions (*n* = 25) occurred in unclassified regions of PIV while 5 and 2 substitutions occurred in domains I and III, respectively [13,14]. Domain II (including the hydrophobic loop), the fusion peptide, and heptad repeat region B (HRB) lacked residue substitutions in Moskva [13,15]. The hydrophobic loop plays a crucial role interacting with the HN protein for fusion activation, and the region is conserved in all the strains of Paramyxoviridae [13,15]. In the HN protein, the stalk was more conserved than the head, with 23 residue substitutions in the head versus only two substitutions in the middle of the stalk (Figure 3B). The stalk interacts with the F protein for fusion, and residue substitutions in the stalk near the head of the protein are less detrimental than those substitutions further from the head of the protein [16]. Alterations in the F and HN glycoproteins further represented the divergence of Moskva while supporting regions of conservation.

## 3. Conclusions

A novel PIV5 strain (Moskva) was reported, and Moskva is hypothesized to have resulted from accidental cross-contamination of Vero cells by a human experiencing respiratory illness. While PIV5 is a global pathogen, our genetic and phylogenetic analysis indicated greater diversity of PIV5 strains than previously reported. The branching of Moskva suggests distinct PIV5 strains are likely circulating globally undetected. Moreover, Moskva had a noteworthy number of residue substitutions in glycoproteins, as well as shorted M and L genes, indicating considerably greater PIV5 diversity than was previously known. Further research is required to understand the implications of PIV5 global presence, the existence of the SH protein, genomic substitutions, and protein truncations (M and F), especially as these topics pertain to human health.

## Figures and Tables

**Table 1:** Comparison of the nucleotide (nt) and amino acid (aa) lengths and identity for PIV5 strains.

Gene/Genome	Length		Excluding Moskva *n* = 26		Including Moskva *n* = 27		Human *n* = 7		Simian *n* = 2		Swine *n* = 4		Bovine, Panda, Pangolin, Tiger *n* = 6	
	nt	aa	nt	aa	nt	aa	nt	aa	nt	aa	nt	aa	nt	aa
Nucleocapsid (N)	1530	509	95.8–100	97.7–100	86.3–100	93.5–100	86.8–87.5	**94.1**–**95.1**	**87.8**	94.5	87.3–87.7	93.9–94.7	87.5–87.7	93.7–94.5
V (V)	669	222	95.5–100	94.6–100	87.3–100	94.6–100	**87.7**–**88.8**	94.6–96.9	**88.8**	**97.3**	87.7–88.3	95.5–96.9	87.4–88.2	95.5–96.9
Phosphoprotein (P)	1177	391	95.8–100	95.9–100	87.1–100	94.1–100	**88.1**–**88.6**	94.4–95.9	**88.6**	**96.2**	87.8–88.3	94.6–95.7	87.9–88.2	94.9–95.7
Matrix (M)	1134 *	377 *	95.3–100	96.0–100	86.2–100	93.7–100	**87.0**–**87.7**	**95.0**–**96.6**	87.4–87.5	95.5–95.8	87.1–87.4	95.5–95.8	86.6–87.4	94.4–95.8
Fusion (F)	1656 ^†^	551 ^†^	94.8–100	95.9–100	83.3–100	91.5–100	**84.9**–**85.8**	**93.3**–**94.6**	85.2	93.7–93.8	85.0–85.1	93.3–93.5	84.9–85.1	93.5–93.7
Hemagglutinin-neuraminidase (HN)	1698	565	95.6–100	95.1–100	84.8–100	91.3–100	85.5–85.8	**92.8**–**94.5**	85.6–85.7	92.6	85.6–85.8	91.9–92.6	84.9–85.8	92.2–92.8
Large (L)	6678 ^‡^	2256 ^‡^	96.9–100	98.1–100	87.4–100	96.9–100	**87.9**–**88.2**	**97.3**–**97.8**	**88.2**	97.5	87.9–88.0	96.9–97.3	**87.8**–**88.2**	97.2–97.6
Entire genome	15,246^§^		95.5–100		85.4–100		**86.2**–**86.7**		**86.6**–**86.7**		86.2–86.4		86.4–86.5	

* Moskva: 1107 nt and 368 aa. ^†^ Simian strains: 1589 nt and 529 aa; Human MEL strain: 1605 nt and 534 aa. ^‡^ Moskva: 6735 nt and 2245 aa. ^§^ Moskva: 15,218 nt. ^¶^ Boldface indicates the highest percent identity to Moskva in the corresponding gene/genome.

## Supplementary Materials

The following are available online at https://www.mdpi.com/1999-4915/11/9/777/s1, Figure SA–H: Individual gene phylogenetic trees of PIV5 strains.

## About the Author

Brandi J. Feehan is a master’s student within the Veterinary Biomedical Sciences program under Douglas Marthaler at Kansas State University. Her primary research objective is to employ bioinformatics to elucidate viromes, resolve quasispecies, and determine relationships amongst different viral populations infecting animals.
