# Supplementary material for: Novel Mammalian orthorubulavirus 5 Discovered as Accidental Cell Culture Contaminant
Source: Viruses. 2019 Aug 23;11(9):777. doi: 10.3390/v11090777 (PMC6783914; doi:10.3390/v11090777)
Supplement: Supplementary file 1 [file viruses-11-00777-s001.zip › viruses-563475-supplementary/viruses-563475-Supplemental Material and Methods.docx]

**Supplemental Materials and Methods**

**Origin of Samples**

Fecal swabs were collected from three-day old piglets during November of 2015 from a farm within the Republic of Buryatia, Russia (Alekseev et al., 2018).

**Cell Culture**

Cell culture occurred as previously described using 1:10 dilution of centrifuged fecal samples to minimal essential medium (MEM) supplemented with 1% actinomycin and 1% non-essential amino acids (Gibco, Grand Island, NY, USA) (Alekseev et al., 2018). The samples were sequentially filtered using 0.8μm, 0.45μm, and 0.2μm, followed by serial dilutions onto Vero cell monolayers. Cell culture supernatants were stored at -70°C. Viral titers were tested on the fourth passage as previously described (Reed and Muench, 1938).

**Extraction**

Nucleic acid was extracted using the GeneJET Viral DNA/RNA Purification Kit with manufacturer protocols (Thermo Scientific, Waltham, MA, USA).

**Library Preparation and Sequencing**

Library preparation occurred using protocols previously described with the initial cDNA created through RevertAid H Minus First Strand cDNA Synthesis Kit (Thermo Scientific, Waltham, MA, USA) (Alekseev et al., 2018). The TruSeq Stranded Total RNA Library Prep Kit, with an input of 1μg, was utilized for library preparation and amplification (Illumina, San Diego, CA, USA). Ribosomal RNA (rRNA) was depleted using the standard protocols of Ribo-Zero rRNA Removal Kit (mixture 1:1 Human/Mouse/Rat probe and Bacteria probe) (Illumina, San Diego, CA, USA). Sequencing occurred on an Illumina HiSeq2000 (Illumina, San Diego, CA, USA).

**Read Trimming**

Sequencing reads were trimmed to remove adapter sequences, indexes, and regions of low phred quality (<20) using Trimmomatic v0.33 as previously described (RWTH Aachen University, Aachen, Germany) (Knutson et al., 2017).

**Read Classification**

Trimmed reads were classified by Kraken with a k-mer size of 24 bases, and a custom database as previously described (John Hopkins University, Baltimore, MA, USA) (Knutson et al., 2017).

***De novo* Assembly**

Reads classified as viral and unknown by Kraken underwent *de novo* assembly by a5 (University of California-Davis, Davis, CA, USA), iva (Wellcome Sanger Institute, Hinxton, England) and ray assemblers (CHUQ Research Center, Quebec, Canada).

**BLASTn**

Assembled contigs were sent through a BLASTn search to elucidate viral contigs (National Center for Biotechnology Information, Bethesda, MD).

**PCR and Gel Electrophoresis**

Primers (forward, CCGGATCACGTGTCCTCAAA; and reverse, ACCAGGAACCGCACTAATGG) specific to Moskva were created within the hemagglutinin-neuraminidase gene. Samples tested for Moskva presence by PCR were the original Vero cell stock, fecal samples, and the 15^th^ cell culture passage. The True-Start DNA polymerase with 10 mM dNTPs mix with manufacturer protocols was used for PCR (Thermo Scientific, Waltham, MA, USA). Gel electrophoresis was completed using the FastRuler Low Range DNA Ladder to determine presence or absence of the 147 base band (Thermo Scientific, Waltham, MA, USA).

**Alignment**

On Geneious version 11.5.1 (Geneious, Newark, NJ, USA), standard MAFFA nucleotide and amino acid alignments, of the complete genome and individual genes, were generated between Moskva and 27 previously published complete PIV5 genomes on GenBank (National Center for Biotechnology Information, Bethesda, MD).

**Phylogenetic Trees**

Nucleotide alignment were generated using a 500 bootstrapped Maximum Likelihood Tree in Geneious with default parameters (Geneious, Newark, NJ, USA). Tree illustrations were generated in FigTree (Institute of Evolutionary Biology, University of Edinburgh, Edinburgh, Scotland) and Adobe Illustrator (Adobe, San Jose, CA, USA).

**Glycoprotein Models**

Fusion and hemagglutinin-neuraminidase models were created in SWISS MODEL (Swiss Institute of Bioinformatics, Lausanne, Switzerland; F, 4jf7.1; HN, 4wsg.1) and Phyre2 (Imperial College London, London, UK; F, c2b9bA; HN, c4jf7B) with modifications through Chimera (Resource for Biocomputing, Visualization and Informatics, University of California, San Francisco, CA, USA).

**References**

Alekseev, K.P., A.A. Penin, A.N. Mukhin, K.M. Khametova, T.V. Grebennikova, A.G. Yuzhakov, A.S. Moskvina, M.I. Musienko, S.A. Raev, A.M. Mishin, A.P. Kotelnikov, O.A. Verkhovsky, T.I. Aliper, E.A. Nepoklonov, D.M. Herrera-Ibata, F.K. Shepherd, and D.G. Marthaler, 2018: Genome Characterization of a Pathogenic Porcine Rotavirus B Strain Identified in Buryat Republic, Russia in 2015. *Pathogens* **7**, 46, DOI: 10.3390/pathogens7020046.

Knutson, T.P., B.T. Velayudhan, and D.G. Marthaler, 2017: A porcine enterovirus G associated with enteric disease contains a novel papain-like cysteine protease. *J Gen Virol* **98**, 1305–1310, DOI: 10.1099/jgv.0.000799.

Reed, L.J., and H. Muench, 1938: A SIMPLE METHOD OF ESTIMATING FIFTY PER CENT ENDPOINTS. *Am J Epidemiol* **27**, 493–497, DOI: 10.1093/oxfordjournals.aje.a118408.
